# Interplay between mast cells, enterochromaffin cells, and sensory signaling in the aging human bowel

**DOI:** 10.1111/nmo.12842

**Published:** 2016-05-20

**Authors:** Y. Yu, D. M. Daly, I. J. Adam, P. Kitsanta, C. J. Hill, J. Wild, A. Shorthouse, D. Grundy, W. Jiang

**Affiliations:** ^1^ Department of Biomedical ScienceUniversity of SheffieldSheffieldUK; ^2^ Department of Colorectal Surgical UnitNorthern General HospitalSheffield Teaching HospitalSheffieldUK; ^3^ Department of HistopathologyNorthern General HospitalSheffield Teaching HospitalSheffieldUK

**Keywords:** aging, EC cells, human bowel afferent, mast cells

## Abstract

**Background:**

Advanced age is associated with a reduction in clinical visceral pain perception. However, the underlying mechanisms remain largely unknown. Previous studies have suggested that an abnormal interplay between mast cells, enterochromaffin (EC) cells, and afferent nerves contribute to nociception in gastrointestinal disorders. The aim of this study was to investigate how aging affects afferent sensitivity and neuro‐immune association in the human bowel.

**Methods:**

Mechanical and chemical sensitivity of human bowel afferents were examined by *ex vivo* afferent nerve recordings. Age‐related changes in the density of mast cells, EC cells, sensory nerve terminals, and mast cell‐nerve micro‐anatomical association were investigated by histological and immune staining.

**Key Results:**

Human afferents could be broadly classified into subpopulations displaying mechanical and chemical sensitivity, adaptation, chemo‐sensitization, and recruitment. Interestingly human bowel afferent nerve sensitivity was attenuated with age. The density of substance P‐immunoreactive (SP‐IR) nerve varicosities was also reduced with age. In contrast, the density of ileal and colonic mucosal mast cells was increased with age, as was ileal EC cell number. An increased proportion of mast cells was found in close apposition to SP‐IR nerves.

**Conclusions & Inferences:**

Afferent sensitivity in human bowel was reduced with advancing age. Augmentation of mast cells and EC cell numbers and the mast cell‐nerve association suggest a compensatory mechanism for sensory neurodegeneration.

**Abbreviations**
5‐HT (serotonin): 5‐hydroxytryptamine
AITC: allyl isothiocyanate
BK: bradykinin
CAP: capsaicin
CgA: chromogranin A
EC cells: enterochromaffin cells
GAPDH: glyceraldehyde 3‐phosphate dehydrogenase
IBS: irritable bowel syndrome
qPCR: real‐time quantitative polymerase chain reaction
SP: substance P
TPH1: tryptophanhydroxylase1
TRP channels: transient receptor potential
TRPA1: transient receptor potential ankyrin receptor
TRPC: transient receptor potential canonical‐related receptor subfamily
TRPM: transient receptor potential melastatin‐related receptor subfamily
TRPV1: transient receptor potential vanilloid 1 receptor

**Key Points**

- There is evidence that as we age the sensory signals that are generated from the bowel become attenuated and while this is borne out by electrophysiological studies in rodents there is a paucity of mechanistic information from humans.
- By recording directly the afferent impulse traffic in resected human colonic we have demonstrated attenuated nerve activity with age that is associated with concurrent changes in nerve density, enterochromaffin cell number and mucosal mast cells.
- These changes in key components of the gastrointestinal surveillance mechanisms has implications for our understanding of the mechanisms underlying the increased prevalence of GI disorders in the elderly.

The incidence and prevalence of a number of gastrointestinal (GI) tract disorders profoundly increase with age.^1^, ^2^ However, relatively little is known about how aging alters sensory signaling from the gut. Previous studies have shown that in humans, visceral pain perception from the GI tract in response to distension was significantly reduced with age.^3^, ^4^ Moreover, abdominal pain and nausea in response to a standard nutrient challenge test (a test in which volunteers are asked to ingest increasing amounts of a liquid nutrient) is also diminished in the elderly.^5^ Experimental studies with rodents have shown that the numbers of myenteric and submucosal neurones decline with age,^6^, ^7^, ^8^, ^9^ and that sympathetic innervation and vagal nerves deteriorate.^10^ A recent study from our laboratory found that mechanosensation and serotonergic chemosensitvity in the mouse intestine was significantly inhibited with age.^11^ However, there are currently no equivalent human data showing how aging affects sensory signaling from the bowel. The interplay between mast cells, enterochromaffin (EC) cells, and the sensory innervation of the gut wall is a key to normal neuro‐immune signaling. Aberrant signaling between these elements may play a significant role in the etiology of functional GI conditions such as irritable bowel syndrome (IBS). For example, in IBS patients, we already know that mast cell and EC cell numbers are elevated in the colon,^12^ the severity of abdominal pain correlates with increased serotonin release from the EC cell,^13^ and the distance between the mast cells and nerve terminals is reduced and correlates to increased abdominal pain.^14^ Moreover, clinical data show that the mast cell stabilizer, disodium cromoglycate improves IBS symptoms.^15^ While neuro‐immune signaling in the bowel has been well studied, the effect that aging has on the normal interaction between mast cells, EC cells, and sensory nerves has yet to be established.

Our understanding of GI sensory function has depended greatly on rodent studies. However, how this translates to humans remains uncertain. There are many examples of therapeutic agents developed using animal models that have failed in the clinic. For example, alosetron, a 5‐HT_3_ receptor antagonist, was approved for marketing to relieve symptoms of IBS but was withdrawn 9 months later owing to serious life‐threatening adverse effects (U.S. Food and Drug Administration, 2002). A number of other drugs have also failed to show efficacy in clinical trials, such as talnetant (NK3 receptor antagonist), GW876008 (corticotropin‐releasing factor‐1 receptor antagonist), and AZD7371 (5‐HT_1A_ receptor antagonist),^16^, ^17^ despite promising data from animal studies.^18^, ^19^, ^20^ These failures highlight a demand for translational research on human tissue. Although *ex vivo* afferent recordings of the rodent GI tract have been well established, very few studies have looked at afferent signaling in man. To address this, our group recently developed an *ex vivo* gut preparation to record afferent activity from isolated segments of the human bowel. These studies were one of the first to report sensory nerve activity from human tissue.^21^ The aim of this study was to examine patterns of innervation, afferent nerve sensitivity, and mast cells, EC cell localization in the aged human bowel.

## Material and Methods

### Ethical approval and donors

Specimens were obtained from 59 patients (35 male) aged between 24 and 88 with a median age of 67, who had undergone right or left hemicolectomy at the Northern General Hospital NHS Foundation Trust in Sheffield. Specimens were collected from patients with a range of disease phenotypes (summarized in Table S1). Tissues were always taken from the healthy tissue bordering the disease area rather from the area of active disease. This was confirmed by a pathologist at the time of collection. Informed written consent was obtained from each participant. Studies were performed according to the declaration of Helsinki and the BMJ guideline on patient consent to publication. Experimental procedures were approved by the South Humber Research Ethics Committee (REC ref: 07/Q1105/4) and the Research Department in Sheffield Teaching Hospitals (ref: STH14755).

### Extracellular afferent nerve recording

Extracellular afferent recording were conducted using an *ex vivo* preparation as previously described.^21^ Forty‐five human bowel specimens primarily from ileum and sigmoid colon were used. Segments were opened longitudinally along the mesentery border and pinned flat with mucosa uppermost in a tissue bath (volume ~100 mL) constantly perfused with gassed (5% CO_2_ and 95% O_2_) Krebs buffer (composition, in mM: NaCl 120, KCl 5.9, MgSO_4_ 1.2, NaH_2_PO_4_ 1.2, NaHCO_3_ 15.4, glucose 11.5, and CaCl_2_ 1.2) at 34 °C. Nerve bundles were identified in the mesentery and drawn into a glass suction electrode attached to a Neurolog headstage (NL100; Digitimer Ltd, Hertfordshire, UK). Afferent signals were amplified (NL104), filtered (NL125 band pass filter), and recorded on a computer via a power 1401 analog‐to‐digital interface and Spike 2 software (Version 7; Cambridge Electronic Design, Cambridge, UK).

After an equilibration period, the receptive field of each afferent was identified by systematically probing the tissue using a glass probe. Mechanosensitivity was examined by applying three stimuli: probing (with calibrated von Frey hair 4, 10, and 60 g; each probing was applied for 2 s and repeated at least 5 times with a 3‐s interval), fine mucosal stroking (1 or 4 g force), and stretch (using forceps). Chemosensitivity was then examined by applying selective agonists directly onto the receptive fields using a pipette. Single unit analysis was performed offline using the spike sorting function of Spike 2 to discriminate the afferent nerve activity of individual units. Afferent activity is expressed as frequency per second (impulse/s).

### Histology and immunohistochemistry

Human bowel specimens were fixed in 4% PFA overnight at 4 °C. For histological staining, fixed specimens were dehydrated in graded ethanol (70%, 90%, 100%, and dried 100%) prior to infiltration in freshly made catalyzed JB4 resin solution. Specimens were then embedded in JB4 resin with accelerator, mounted on aluminum stubs and sectioned at 2 *μ*m using a microtome (LKB 2218; LKB‐Produkter, Bromma, Sweden). For immunohistochemistry, fixed tissue was cryo‐protected with 30% sucrose overnight at 4 °C, embedded in OCT (53581; Bright Instrument Company, Huntingdon, UK), and sectioned at 10 *μ*m using a cryostat (Bright Instrument Company).

### Toluidine blue staining

Sections were stained with 0.1% toluidine blue for 3 min and mounted with coverslips using DPX mounting medium. Three populations of mast cells (mucosa, submucosa, and serosa) were blindly quantified under a 20× objective with an Olympus BX51 (Tokyo, Japan) light microscope using an eyepiece graticule (area: 0.35 mm × 0.35 mm = 0.1225 mm^2^). Ten random microscope fields of mucosa and serosa and 20 random fields of submucosa were quantified for each section. Data were expressed as number of mast cells/mm^2^.

### Immunohistochemical staining

Sections were incubated with a primary antibody for EC cells (rabbit antiserotonin, 1 : 50, [AHP522], AbD Serotec, Oxford, UK) or a mixture of primary antibodies for mast cells (mouse antimast cell tryptase, 1 : 1000, [Sc271095] Santa Cruz, Heidelberg, Germany) and substance P (SP; rabbit anti‐SP, 1 : 50, Abcam, ab24126) overnight at 4 °C. After rinsing with PBS, slides were stained with corresponding secondary antibodies, goat anti‐rabbit conjugated to Cy3 (1 : 400, [111‐165‐144], Jackson ImmunoResearch, West Baltimore Pike, PA, USA) or a mixture of goat anti‐mouse Cy3 (1 : 400, [115‐166‐003] Jackson ImmunoResearch, Baltimore West Pike, PA, USA) and goat anti‐rabbit FITC (1 : 400, [FI‐1000] Vector laboratories, Peterborough, UK), for 2 h at room temperature. Slides were mounted with coverslips using mounting medium (Vector H1200). Negative controls were performed by omitting the primary antibody. Staining was observed under an Olympus BX51 microscope. Images were captured using an Olympus ColorView II digital camera for offline quantification. Images were analyzed using ImageJ software (1.43u; National Institutes of Health, Bethesda, MD, USA) where applicable.

Substance P density was quantified as the area occupied by SP as a percentage of the total area of mucosa. Enterochromaffin cell number was counted and normalized to the area of mucosa. The distance between a mast cell and its nearest SP‐IR varicosity was measured using ImageJ.

As a negative control for mast cell‐SP association, image flipping was performed. Prior to superimposing images for mast cell staining and SP staining, one of the channels was flipped horizontally. Measurement of mast cells‐SP distances and distribution analysis was performed to ensure that proximity was not due to a random occurrence (Fig. S3).

### H&E staining and electron microscopy

See supplementary methods (Data S1).

### PCR

See supplementary methods (Table S2).

### Drugs and compounds

All drugs, chemical compounds, and reagents were purchased from Sigma‐Aldrich (Dorset, UK) unless stated specifically.

### Data analysis and statistics

All data are expressed as mean ± SEM. Statistical significance was confirmed using either a Student's *t*‐test or one‐way anova as appropriate. *p* < 0.05 was considered as significant. To evaluate the influence of aging, a linear regression test was performed. All quantification was performed in a blinded manner. All statistical analysis was performed using GraphPad Prism 5 (GraphPad Software Inc., La Jolla, CA, USA).

## Results

### Human bowel afferent recordings and ultrastructure of recorded nerves

Afferent nerve firing was recorded in 20 of 45 specimens, giving an overall success rate of 45%, of these only 8 specimens were responsive to mechanical or chemical stimulation. Success rate was not dependent on the age of donor or the region of specimens (Fig. S1).

Structural integrity of the bowel wall was preserved with normal morphology revealed by H&E staining (Fig. S2A). Electron microscopy showed the mesenteric nerve bundles to vary between 40 and 90 *μ*m in diameter, containing 1–3 fasciculi enveloped by a thin perineurium. The majority of nerve fibers were unmyelinated, although there were also a few myelinated nerve fibers easily identified by their characteristic dark sheath on the electron micrograph (Fig. S2C).

### Characterization of mechanosensitive human bowel afferents

**Figure 1:**
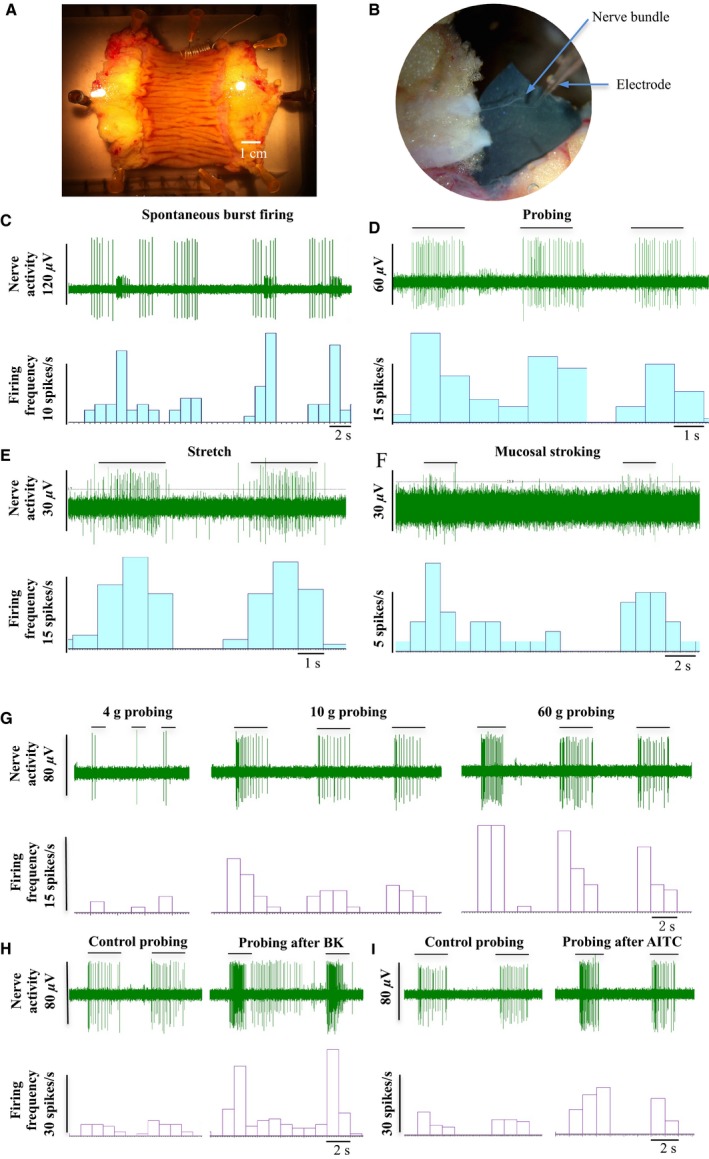
Extracellular afferent nerve recordings of the human bowel. (A and B) Photographs showing a human gut preparation and dissection of a nerve bundle, (C) Representative traces showing spontaneous burst firing (58‐year‐old, ileum), (D) afferent response to probing (77‐year‐old, colon), (E) stretch (77‐year‐old, colon), and (F) mucosal stroking (42‐year‐old, colon). (G) Afferent response to incremental probing with von Frey hairs (67‐year‐old, colon). (H and I) Probing evoked afferent firing before and after treatment with 10 *μ*M bradykinin and 300 *μ*M AITC, respectively (serosal afferent, 67‐year‐old, colon).

A total of 34 afferent units from 20 specimens were discriminated by Spike2 single unit analysis. Ten of these units (29%) were mechanosensitive, differentially responding to probing, mucosal stroking, and stretch, and thus classified into mesenteric, serosal, muscular, and mucosal afferents (Table 1 and Fig. 1). Twenty‐four units did not respond to probing, stroking, or stretch and were termed unclassified.

**Table 1:** Characteristics of human bowel afferent mechanosensitivity

Classification	Number of units (age)	Spontaneous burst firing	Probing	Mucosal stroking	Stretch
Mesenteric afferent	2 (24, same donor)	Yes	Yes	No	No
Serosal afferent	5 (42, 53, 55, 67, 72)	1: yes 4: no	Yes	No	No
Muscular afferent	2 (49, 77)	1: yes 1: no	Yes	No	Yes
Mucosal afferent	1 (42)	Yes	Yes	Yes	No
Unclassified	24 (median 66, range: 24–77)	7: yes 17: no	No	No	No

One serosal unit responded to repeated probing with a gradually reduced firing rate, indicating the nature of mechano‐adaptation. The unit also showed a dramatic increase in firing rate to the incremental probing force at 4, 10, and 60 g applied with a calibrated von Frey hair (Fig. 1G). The mechanosensitivity to probing was enhanced after treatment with 10 *μ*M bradykinin (BK) and 300 *μ*M allyl isothiocyanate (AITC; Fig. 1H and I).

### Chemosensitivity of human bowel afferents and silent afferents

**Figure 2:**
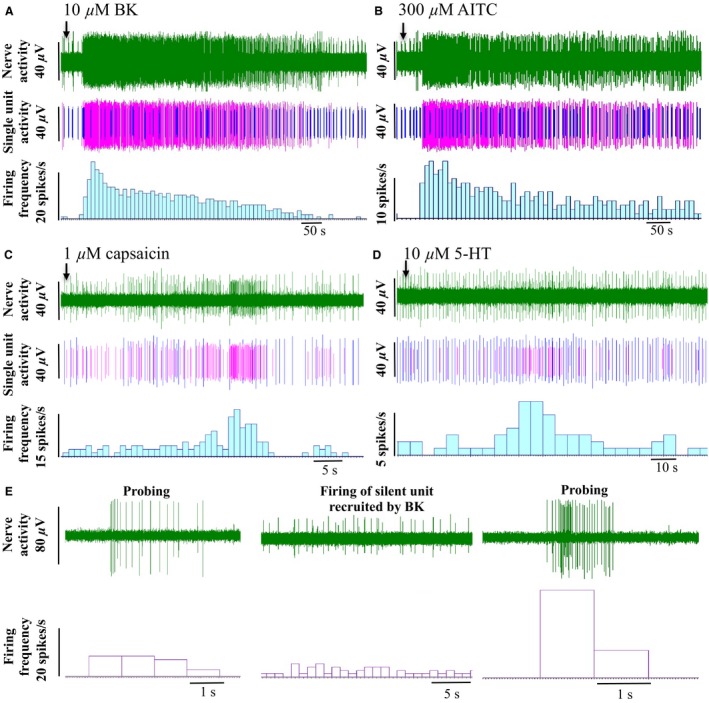
Chemosensitivity of human bowel afferents. Representative traces showing the afferent response to application of (A) 10 *μ*M BK, (B) 300 *μ*M AITC, (C) 1 *μ*M capsaicin, and (D) 10 *μ*M 5‐HT. The top trace of each panel is the raw neurogram, middle trace shows the activity of responding units (purple) and non‐responding units (blue), and the lower trace is the corresponding firing frequency from the responsive units. (E) Representative trace showing the activation of a silent unit following stimulation with BK.

Once the receptive field had been identified, the chemosensitivity was investigated by applying BK (10 *μ*M), capsaicin (CAP; 1 *μ*M), AITC (300 *μ*M) or 5‐HT (10 *μ*M) directly onto the receptive field. Forty percent (4/10) of mechanosensitive units responded to either one or two chemical stimuli (Table 2 and Fig. 2). Interestingly two colonic afferent units (one from a 49, the other from a 67‐year‐old donor) that did not show any firing activity before chemical stimuli were recruited and started to fire after BK treatment (Fig. 2E). These units were classified as ‘silent’ afferents. However, they did not acquire mechanosensitivity after becoming active. Four units (2 serosal and 2 silent) showed a significant response to BK (basal *vs* peak firing rate: 0.2 ± 0.1 *vs* 1.9 ± 0.5 impulses/s, *p* < 0.05, paired *t*‐test). The two BK‐sensitive serosal units also responded to AITC (basal *vs* peak firing rate: 0.3 ± 0.2 *vs* 1.4 ± 0.1 impulses/s). Two units (one serosal, the other muscular) responded to CAP (basal *vs* peak firing: 0.7 ± 0.7 *vs* 4.2 ± 2.7 impulses/s). The muscular unit also responded to 5‐HT (basal *vs* peak: 0.4 *vs* 1.6 impulses/s).

**Table 2:** Characteristics of human bowel afferent chemosensitivity

Classification	Responsive unit (age)	Drugs			
		BK (10 *μ*M)	CAP (1 *μ*M)	AITC (300 *μ*M)	5‐HT (10 *μ*M)
Serosal afferent	1 (55)	Yes	No	Yes	No
	1 (42)	Yes	No	Yes	NT
	1 (53)	No	Yes	No	NT
Silent afferent	1 (67)	Yes	No	No	NT
	1 (49)	Yes	No	No	NT
Muscular afferent	1 (77)	NT	Yes	NT	Yes

NT, not tested.

### Age‐related changes in human bowel afferent sensitivity

**Figure 3:**
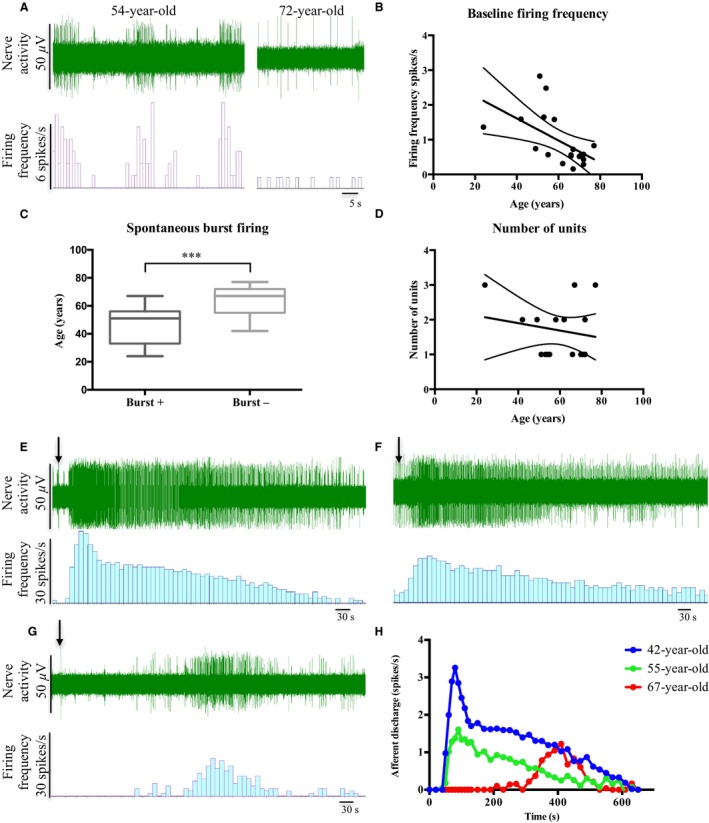
Human bowel afferent sensitivity was altered with age. (A) Representative traces of baseline firing in specimens from a 54‐ and 72‐year‐old donor. (B) Average baseline firing frequency was significantly decreased with age (*p* < 0.05, linear regression, *r* ^2^ = 0.304, *N* = 19). (C) The donor age of the afferent units displaying spontaneous burst firing was significantly lower than that without burst firing (*p* < 0.001, unpaired *t*‐test, *N* = 13 for burst+, *N* = 23 for burst‐). (D) Number of units recorded was not significantly changed with age (*p* = 0.489, linear regression, *r* ^2^ = 0.029, *N* = 19). (E–H) Afferent responses to bradykinin appeared to be decreased with age in the human sigmoid colon. (E) Representative response to 10 *μ*M bradykinin from a 42‐year‐old donor, (F) Representative response to 10 *μ*M bradykinin from a 55‐year‐old donor, and (G) Representative response to 10 *μ*M bradykinin from a 67‐year‐old donor. (H) Response to bradykinin from preparations from 3 donors. ***denotes significance at *p* < 0.001.

Resting nerve activity was present in all preparations, although a small proportion of units did not show basal activity. With increasing age, average baseline firing frequency was significantly decreased (Fig. 3B, *p* < 0.05, linear regression, *r* ^2^ = 0.304, *N* = 19). In 35% (12/34) of units, baseline firing exhibited a bursting pattern (spontaneous burst firing). This was also correlated with age such that the preparations showing burst firing were from significantly younger donors (mean age 47.2 ± 4.1, *N* = 13) than preparations without burst firing (mean age 63.9 ± 2.2, *N* = 23, Fig. 3C, *p* < 0.001, unpaired *t*‐test). However, the number of recorded units per bundle was not significantly altered by age (Fig. 3D, *p* = 0.489, linear regression, *r* ^2^ = 0.029, *N* = 19). The afferent response to 10 *μ*M bradykinin was examined in preparations from the sigmoid colon of a 42‐, 55‐, and 67‐year‐old donor. In each case, bradykinin induced a robust increase in nerve activity, however, the response profile was blunted with increased age (Fig. 3E–H).

### Afferent nerve density decreased in the aged human colonic mucosa

**Figure 4:**
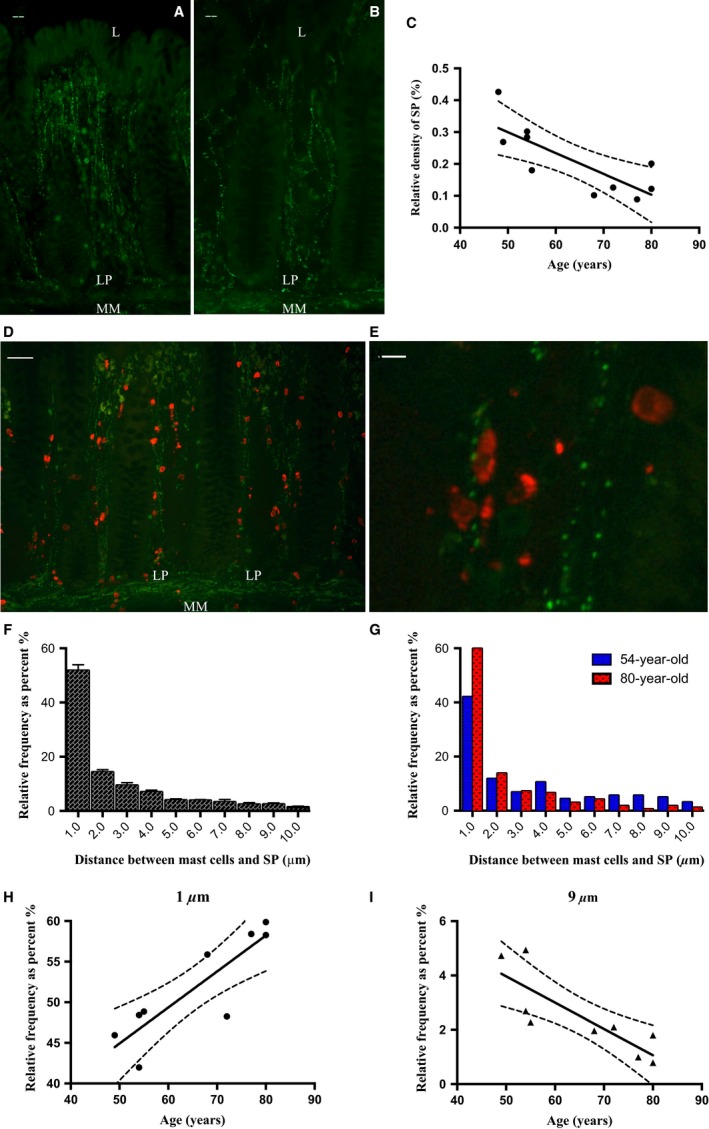
Density of SP‐positive nerve fibers and the mast cell‐SP anatomical relationship in the human colonic mucosa. (A and B) representative images showing SP‐IR (green) nerve fibers running through the laminar propria of colonic mucosa (54‐ and 80‐year‐old donors, respectively). (C) Density of SP in colonic mucosa was reduced with advanced age (*p* < 0.01, *r* ^2^ = 0.614, linear regression, *N* = 10). (D) Representative image showing tryptase‐positive mast cells (red) and SP‐IR varicosities (green) in the colonic mucosa. (E) Magnified image showing the close apposition between mast cells and SP. (F) Frequency distribution pattern of distances between mast cell and SP (*N* = 9). (G) A representative comparison of the frequency distribution patterns in 54‐ and 80‐year‐old donors. (H) The percentage of mast cells within 1 *μ*m distance to SP was increased with age (*p* < 0.01, *r* ^2^ = 0.741, linear regression, *N* = 9) but reduced within 8–9 *μ*m distance (I: *p* < 0.01, *r* ^2^ = 0.684, *N* = 9). LP, laminar propria; MM, muscularis mucosae. Scale bar = 20 *μ*m (A, B), 50 *μ*m (D), 10 *μ*m (E).

Afferent nerve fibers were labeled with the sensory neuronal marker SP. Positive staining was detected in the mucosa, submucosa, myenteric plexus, and in close vicinity to blood vessels. Substance P‐immunoreactive nerve fibers were extensively distributed in the lamina propria of mucosa, but not in the submucosa and muscle layers. The density of mucosal SP was significantly reduced with age (Fig. 4C, *p* < 0.01, linear regression, *r* ^2^ = 0.614, *N* = 10).

### Closer association between mast cells and afferent nerves with age

The micro‐anatomical relationship between mast cells and afferent nerve terminals in the human colonic mucosa was investigated by double immunostaining with mast cell tryptase and SP antibodies (Fig. 4D and E). Analysis of proximity showed that more than 50% of mast cells were located within 1 *μ*m from a SP‐IR varicosity (Fig. 4F). Comparison of distribution patterns between young and aged donors suggested that an increased proportion of mast cells were in close apposition to SP‐IR varicosities in aged samples (Fig. 4G–I).

### Mast cell density increased in the aged mucosa

**Figure 5:**
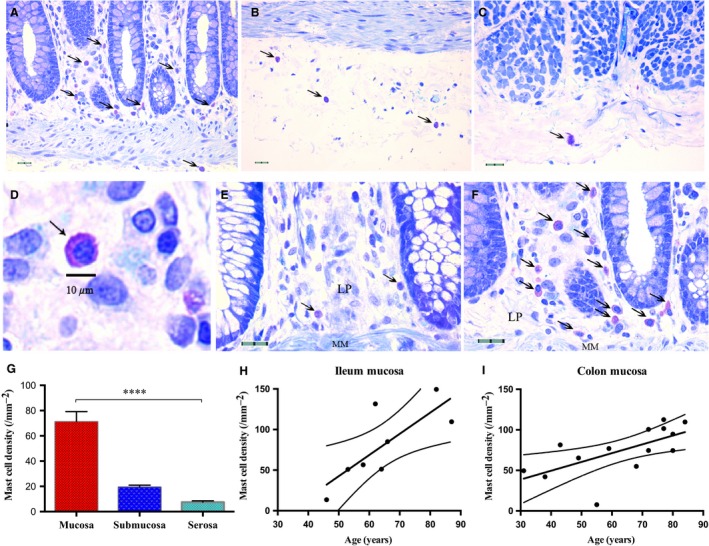
Age‐related changes in human bowel mast cell density. Representative images showing toluidine blue staining in the (A) mucosa, (B) submucosa, (C) serosa. (D) A magnified image showing a mucosal mast cell. (E and F) Representative images of colonic mucosal mast cells from young and aged donors (43‐ *vs* 84‐year‐old). (G) Mucosal mast cells density was much greater than that in the submucosa and serosa in the colon (*p* < 0.0001, one‐way anova,* N* = 15). (H and I) Mucosal mast cell density in both ileum (*p* < 0.05, linear regression, *r* ^2^ = 0.606, *N* = 8) and colon (*p* < 0.05, *r* ^2^ = 0.413, *N* = 14) was positively correlated with age. LP, laminar propria; MM, muscularis mucosae. Scale bar = 10 *μ*m in D; 20 *μ*m in A–C, E, and F. ****denotes significance at *p* < 0.0001.

Mast cells stained with toluidine blue were identified by their red‐purple cytoplasmic metachromatic granular content and blue nuclei (Fig. 5A–F). Mast cells were detected throughout the human bowel wall with a greater density close to the muscularis mucosa. Occasionally some positive cells were also identified in the connective tissue and blood vessels running through muscle layer. The density of mucosal mast cells was significantly higher than in the submucosa and serosa in both small intestine (*p* < 0.001, one‐way anova,* N* = 9) and colon (*p* < 0.0001, *N* = 15, Fig. 5G). Mucosal but not submucosal or serosal mast cell density was positively correlated with increased age in both the ilium (Fig. 5H, *p* < 0.05, *r* ^2^ = 0.606, linear regression, *N* = 8) and large intestine (colon and rectum; Fig. 5I, *p* < 0.05, *r* ^2^ = 0.413, *N* = 14).

### EC cell density increased in aged human ileal mucosa

**Figure 6:**
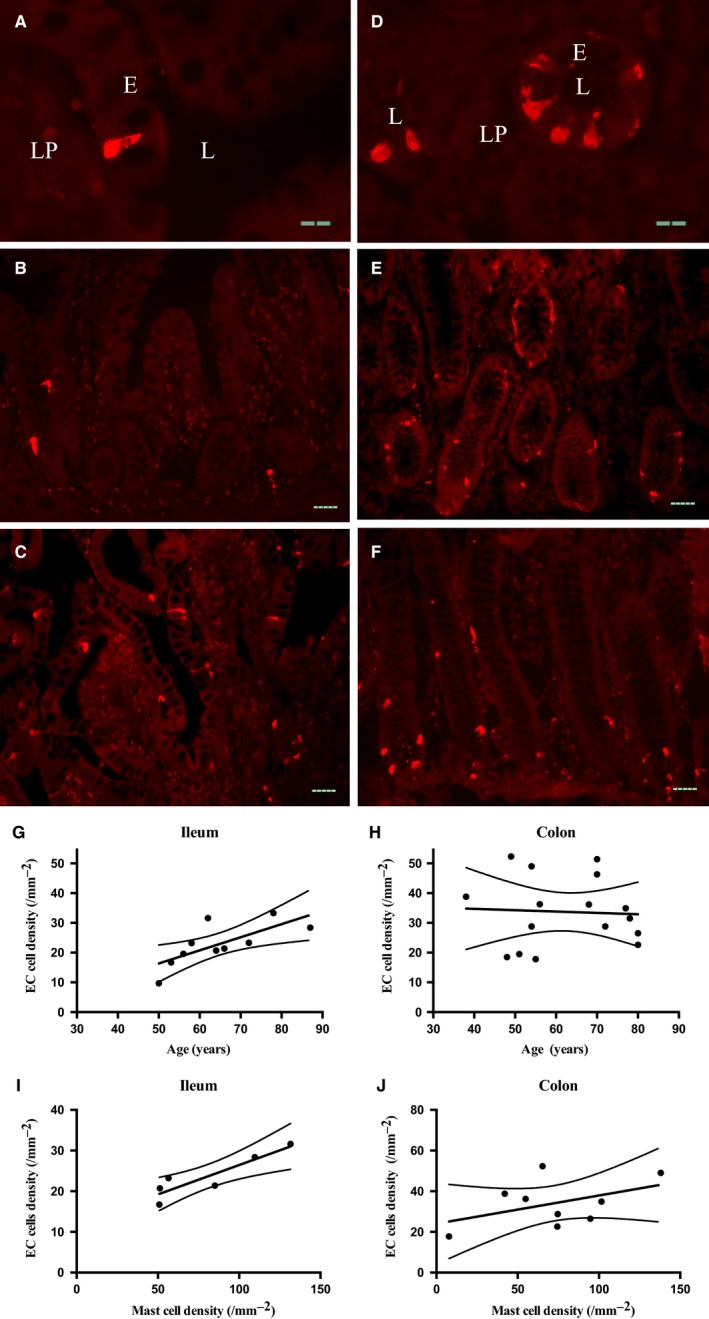
Age‐related changes in human bowel EC cell density. (A) Representative images showing a lateral view of an EC cell in a villus of ilium. (B and C) EC cells detected in the ileal mucosa from young (B, 50‐year‐old) and aged donors (C, 87‐year‐old). (D) Representative image showing a topical view of EC cells surrounding colonic pits. (E and F) Representative image showing EC cells in the colonic mucosa from a young (E, 49‐year‐old) and an aged donor (F, 80‐year‐old). (G and H) EC cells density in the ileum (G, *p* < 0.05, *r* ^2^ = 0.528, linear regression, *N* = 10) but not colon (H, *p* = 0.848, *r* ^2^ = 0.003, *N* = 16) was increased with age. (I and J) Consistently, EC cell density also showed positive correlation with mast cell density in the ileum (I, *p* < 0.05, *r* ^2^ = 0.818, *N* = 6) but not colon (J, *p* = 0.226, *r* ^2^ = 0.201, *N* = 9). E, epithelium; L, lumen; LP, laminar propria. Scale bar = 20 *μ*m in A and D; 50 *μ*m in B, C, E, and F.

Serotonin immunoreactivity was used as a marker for EC cells. Positive cells had a classical conical shape and were relatively concentrated in the small and large intestinal crypts (Fig. 6A–F). Density in the ileum (*p* < 0.05, *r* ^2^ = 0.528, linear regression, *N* = 10), but not colon (*p* = 0.848, *r* ^2^ = 0.003, *N* = 16) was positively correlated with advancing age. Consistently, serotonin immunoreactivity was also correlated with mast cell density in the ileum (Fig. 6I, *p* < 0.05, *r* ^2^ = 0.818, *N* = 6) but not colon (Fig. 6J, *p* = 0.226, *r* ^2^ = 0.201, *N* = 9).

### Gene expression in the aged human bowel

Gene expression for 7 transient receptor potential (TRP) channels (TRPA1, TRPC4, TRPC6, TRPM2, TRPM4, TRPM8, TRPV1) and 2 EC cell markers (Chromogranin A, CgA and tryptophanhydroxylase1, TPH1) was examined using PCR. The highest expression was found for TRPA1, TRPM4, and CgA. The expression of TRPA1 and TRPV1 in the ileum was significantly greater than in the colon suggesting regional variation, however, when expression in young tissues (<65 years old) was compared to expression in older tissues (>65 years old) no significant difference was seen (Fig S4).

## Discussion

This study provides the first evidence that sensory firing from the human bowel is attenuated with age. Attenuation was concurrent with a decrease in the sensory nerve marker SP, but an increase in mast cell and EC cell numbers. Interestingly, the anatomical proximity of mast cells and SP expressing afferent nerves was increased. Together these data suggest that aging alters sensory and neuro‐immune signaling in the human gut.

### Characterization of human bowel afferent nerves and changes associated with aging

To date very few studies have attempted to record from human afferents. In the first part of this study, we conducted a series of afferent nerve recordings using human tissues. Our first task was to characterize the afferent nerve phenotype in man and investigate how this changes as a result of age.

Many previous studies have investigated afferent signaling using *in vivo* and *in vitro* animal models, this has led to a classification of afferents depending on their sensitivity to mechanical stimuli and the location of the receptive fields. Previously, mechanosensitive nerves have been classified into five subpopulations: mesenteric, serosal, muscular, mucosal, and muscular/mucosal afferents using colonic tissues from the rat^22^ or mouse.^23^, ^24^ In this study using mechanical stimulus as a method of classification (probing, stretch, and mucosal stroking), the same subpopulations were identified. Interestingly, we also identified a high proportion of mechanically insensitive afferents (71%). This proportion was much higher than what has previously been reported for pelvic (23%) and lumbar splanchnic (33%) afferents innervating mouse colorectum^24^ suggesting that while some functional characteristics of gut afferents might be species independent, there might still be some species‐related differences between human and mouse that remain to be identified.

In our characterization, we also identified two mechanically insensitive units which were recruited following application of bradykinin. This type of afferent has previously been identified as a silent nociceptor, a fiber, which only fires following sensitization after exposure to an inflammatory mediator.^24^, ^25^ A previous systematic study revealed that about 30% of afferents innervating the mouse colorectum are normally mechanically insensitive, but a proportion of them acquire mechanosensitivity after application of an inflammatory soup.^24^ Silent afferents may be important contributors to the development and maintenance of hypersensitive states; therefore, identifying them and characterizing them in the human might have significance to understanding the pathogenesis of many GI disorders.

We also observed other functional characteristics that have only previously been demonstrated in animals.^23^, ^26^, ^27^ These included graded responses to probing, fast adaptation to repeated stimulation and mechanical sensitization induced by bradykinin, and the TRPA1 agonist AITC. A proportion of the afferent units that we recorded exhibited chemosensitivity, responding to CAP, bradykinin, AITC, or 5‐HT. Interestingly, four of these chemosensitive afferent units also responded to mechanical stimuli, something characteristic of a polymodal nociceptor. Unfortunately due to tissue availability, extensive characterization of human bowel afferents was not feasible. However, it is clear that many of the functional characteristics previously identified in animals were also present in our human bowel recordings.

In a recent study from our laboratory the effect of aging on mouse afferent nerve sensitivity was measured. We found that aging correlated with a reduction in sensory firing and altered serotonergic signaling.^11^ A similar decline in afferent sensitivity was also observed for the human bowel although sample size was limited by the availability of tissue. Basal nerve activity was reduced with age, less burst firing was seen in preparations from older people and sensitivity to the inflammatory mediator bradykinin was blunted. While this is by no means conclusive evidence, these data do support the concept that aging is associated with reduced sensory signaling. This may contribute to reduced pain signaling in the elderly.

Previous morphological studies suggest that neuronal numbers in the enteric nervous system decline with age.^6^, ^9^, ^28^, ^29^ Sympathetic and vagal innervation in the gut also show signs of degeneration^10^, ^30^ and a reduction in the size or number of primary neurons in the mammalian nodose and dorsal root ganglia (DRG) have been described.^31^ In this study, we measured sensory nerve density using SP as a marker, and found significant attenuation in immunoreactivity in correlation with increasing age. Two previous studies also reported a decline in SP‐positive nerve fibers in the circular muscle of the aged guinea‐pig colon^32^ and the DRG of the aged rat.^33^ While we recognize that this is just one marker of afferent nerve endings, these data suggest that either neuropeptide content in the afferent terminal is reduced with age or that aging induces neurodegeneration in the gut wall. Clearly further studies are still required to fully identify how aging alters innervation patters in the bowel, however, sensory neurodegeneration may explain the decreased sensitivity observed in our afferent nerve recordings.

### Neuro‐immune interactions and aging

Altered neuro‐immune interactions might also play a role in changing afferent sensitivity in the aged bowel. Our data revealed an age‐related increase in the density of mucosal mast cells and EC cells in the gut wall. It has been proposed previously that increased circulating levels of pro‐inflammatory cytokines including interleukin (IL)‐1, IL‐6, tumor necrosis factor‐alpha, and IL‐12, interferon‐alpha/beta promote a low‐grade chronic systemic pro‐inflammatory state in the elderly.^34^, ^35^, ^36^ This state is associated with enhanced immune cell infiltration and could explain the increase in mast cell numbers observed in this study.

Previous studies have reported an anatomical association between mast cells and SP‐positive nerves in a variety of rodent organs, human skin, and the colon of IBS patients.^37^, ^38^, ^39^, ^40^ Interestingly, when we looked at the proximity of mast cells to the SP‐positive fibers in our human tissues, we found a significant correlation with age, whereby mast cells were in closer association with the nerve fibers in tissues from older donors compared to those from younger donors. In our analysis, we normalized the absolute number of cells to the relative distribution excluding the possibility that this closer association was only secondary to increased cell number. Why this mast cells‐nerve association is altered with age remains unclear, however, it is tempting to speculate that with aging, mast cells could migrate toward the nerve fibers as a compensatory mechanisms due to a reduction in innervation.

The EC cell, releases 5HT to regulate motility, secretion, and neuronal activity. In our previous study looking at the effect of aging in the mouse intestine, we found EC cell numbers were elevated but that afferent sensitivity to 5‐HT was reduced. In this study, numbers of EC cells in the ileum but not the colon were also elevated with age. As with the increased mast cell number, increased EC cell density might also be a consequence of accumulation of inflammatory events. It has been suggested that EC cell hyperplasia and reduced serotonin transporter (SERT) expression may persist in the gut as a consequence of short‐term inflammation.^41^ Indeed, in this study, we found a positive correlation between mast cell number and the density of EC cells in the ileum. However, our PCR data found that the expression of TPH1 was not changed suggesting that 5HT bioavailability is not altered with age.

### Limitations and advantages of using human tissue

The vast majority of what we know about the function of the gut has been gained from animal studies, however, very little translational research looking at human GI physiology has been conducted. In this study, we wanted to investigate how afferent signaling and neuro‐immune function was affected by age in human tissues. Although studies on human tissues offer the distinct advantage of looking directly at the function of the human bowel, key disadvantages and limitations exist. Firstly, conducting the *ex vivo* extracellular afferent recordings using human tissues proved to be extremely technically challenging. In a typical recording of mouse jejunum, 10–15 afferent units can be simultaneously recorded, however, in human tissues, the recording sites were very close to the gut wall and typically only 1 or 2 units were recorded. Secondly, the region of the gut from where the tissues were taken was unpredictable (i.e. ileum, colon, or rectum) and tissue variability was an issue, finally variations in donor age, gender, genetic background, and medical history add complexity to the data. As previous studies have revealed alterations in mast cell, EC cells, and SP with IBS, understanding donor symptomology might also influence the evaluation of age‐related changes in the gut. Although our specimens were taken from the ‘safe margins’ and appeared to be morphologically normal, the changes we observed could have been influenced by disease phenotype. Also outside our control was any ongoing or previous treatment patients may have been receiving.

## Conclusions

This study is the first to provide insight into the functional and morphological changes in the sensory function of human bowel with age. Although reliant on relatively small sample sizes, and taking into consideration all the limitations described, we find that the aged bowel has reduced afferent sensitivity and increased immune cell numbers suggesting altered neuro‐immune function occurs with aging. Our findings clearly show some key alterations in the human gut. However, further studies are now required to provide detailed mechanistic insight.

## Funding

This project was funded by the Bowel Disease Research Foundation (BDRF), Sheffield Hospital Trust (SHT) and Biotechnology and Biological Sciences Research Council (BBSRC).

## Conflicts of Interest

Authors declare no conflict of interest.

## Author Contribution

YY performed the experiments; WJ obtained H&E images; WJ and DG designed the research study and obtained funding; IA, PK, JW, and AS consented patients and helped with the collection of donor samples; CH performed electron microscopy; YY and WJ analyzed the data; YY, WJ, and DD produced the figures and wrote the manuscript; DG and WJ are the joint corresponding authors.

## Supporting information


**Table S1** Table showing the disease phenotype of specimens used in this study and the experiments they were used for.
**Table S2** Fluorescent probes sequences used in real‐time quantitative PCR.
**Figure S1** Correlation between success rate of a recording and the age and region of specimens.
**Figure S2** Tissue integrity and ultrastructure of recorded nerves.
**Figure S3** The association between mast cells and SP was not random.
**Figure S4** Changes in TRP channel and EC cell gene expression in the human bowel.
**Data S1** Supplementary methods.
