# Supplementary material for: Interplay between mast cells, enterochromaffin cells, and sensory signaling in the aging human bowel
Source: Neurogastroenterol Motil. 2016 May 20;28(10):1465–79. doi: 10.1111/nmo.12842 (PMC5053273; doi:10.1111/nmo.12842)
Supplement: Supplementary file 1 — Table S1 Table showing the disease phenotype of specimens used in this study and the experiments they were used for. Table S2 Fluorescent probes sequences used in real‐time quantitative PCR. Figure S1 Correlation between success rate of a recording and the age and region of specimens. Figure S2 Tissue integrity and ultrastructure of recorded nerves. Figure S3 The association between mast cells and SP was not random. Figure S4 Changes in TRP channel and EC cell gene expression in the human bowel. Data S1 Supplementary methods. [file NMO-28-1465-s001.docx]

# Supporting information

**Supplementary table S1**

Table showing the disease phenotype of specimens used in this study and the experiments they were used for

| **Tissue segment** | **Adenoma /carcinoma** | **Cohn’s disease** | **Colitis** | **Diverticular disease** | **Perforated appendicitis** | **Proctitis cystica profunda** |
| --- | --- | --- | --- | --- | --- | --- |
| **Ileum** | 11 (5/9R, 8M, 8E, 5P) | 5 (1R, 1M, 2E, 1P) |  |  |  |  |
| **Sigmoid colon** | 18 (5/10R, 6M, 7E, 2SP, 7P) |  | 1 (0R, 1 E, 2SP ) | 3 (0/2R,1M, 2E, 2SP ) |  | 1(0R, 1M, 1E) |
| **Ascending colon** | 6 (2/4R, 4M, 3E, 3SP, 1P) |  | 1(1M ) |  | 1(0R) |  |
| **Transverse colon** | 2 (0R, 1M, 1E, 1SP, 1P) |  |  |  |  |  |
| **Descending colon** | 7 (5R, 1M, 1E, 1SP, 6P) |  |  |  |  |  |
| **Rectum** | 3 (1/2R, 1M) |  |  |  |  |  |
| **Totals** | **47 (79.6%)** | **5 (8.5%)** | **2 (3.4%)** | **3 (5.1%)** | **1 (1.7%)** | **1 (1.7%)** |

(R, successful nerve recording; M, mast cell quantification; E, EC cell quantification; SP, SP quantification; P, PCR.) For example: 11 (5/9R) indicates that 9 out of 11 ileum samples from a patient with a tumour were used for nerve recording, 5 of which gave a successful recording.

**Supplementary S2: Fluorescent probes sequences used in real-time quantitative PCR.**

| **Target Genes** | **Accession No.** | **Sequence** | | **Expected Size (bp)** |
| --- | --- | --- | --- | --- |
| TRPA1 | NM_007332 | Forward | 5' GCCACTGAGATTGTTAAACTGATG 3' | 129 |
|  |  | Reverse | 5' GTCTGCTAGCTCATGGTGAT 3' |  |
| TRPC4 | NM_016179 | Forward | 5' CGTCGAGTGGATGATATTACCG 3' | 117 |
|  |  | Reverse | 5' ACAAAGTCCATTAGATTCCACCA 3' |  |
| TRPC6 | NM_004621 | Forward | 5' AGGACTATCTGCTCATGGACT 3' | 113 |
|  |  | Reverse | 5' GTGAGCCAGTCTGTTGTCAG 3' |  |
| TRPM2 | NM_003307 | Forward | 5' TCCTCCTACTCTGCCTCTAC 3' | 111 |
|  |  | Reverse | 5' AAATCTGGTCCGTGTGCTC 3' |  |
| TRPM4 | NM_001195227 | Forward | 5' CAGTGACTTCCCAAGTATCCTG 3' | 111 |
|  |  | Reverse | 5' CAGTTGCTGTGCTCCATGA 3' |  |
| TRPM8 | NM_024080 | Forward | 5' GCTGGCTAATGAGTACGAGAC 3' | 126 |
|  |  | Reverse | 5' TCCAGACAGTTGCTTCCAC 3' |  |
| TRPV1 | NM_080705 | Forward | 5' TCCTGCTCAACATGCTCATC 3' | 132 |
|  |  | Reverse | 5' AGCTCTTCTCCGTGTCCA 3' |  |
| TPH1 | NM_004179 | Forward | 5' CCATTGTGCCAACAGAGTTC 3' | 115 |
|  |  | Reverse | 5' GTTCATAGCCAAGTCCGCAA 3' |  |
| CgA | NM_001275 | Forward | 5' GATCCTTTCCATTCTGAGACATCA 3' | 128 |
|  |  | Reverse | 5' GAACCTCTGAGAGTTCATCTTCA 3' |  |
| GAPDH | NM_002046 | Forward | 5' ACATCGCTCAGACACCATG 3' | 143 |
|  |  | Reverse | 5' TGTAGTTGAGGTCAATGAAGGG 3' |  |

**Supplementary figure 1**

**Supplementary figure 2**


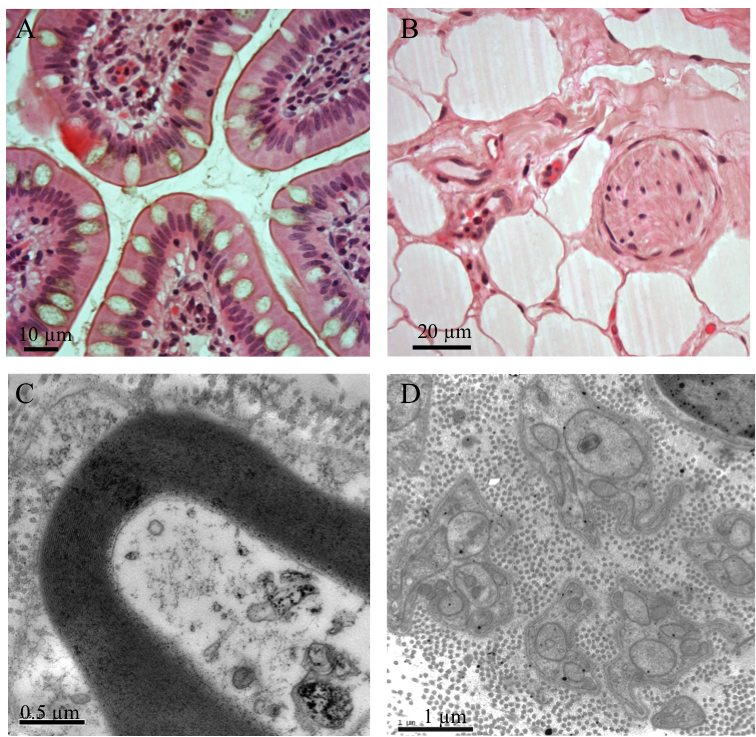


**Supplementary figure** **3**


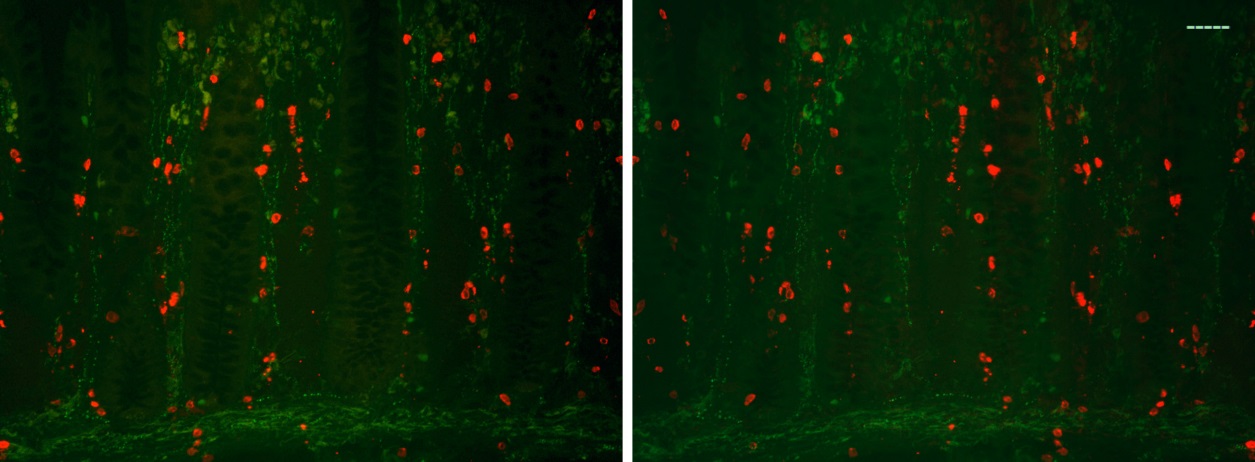


A

B

D

C

Control

Flippedl

SDA

**Supplementary figure 4**


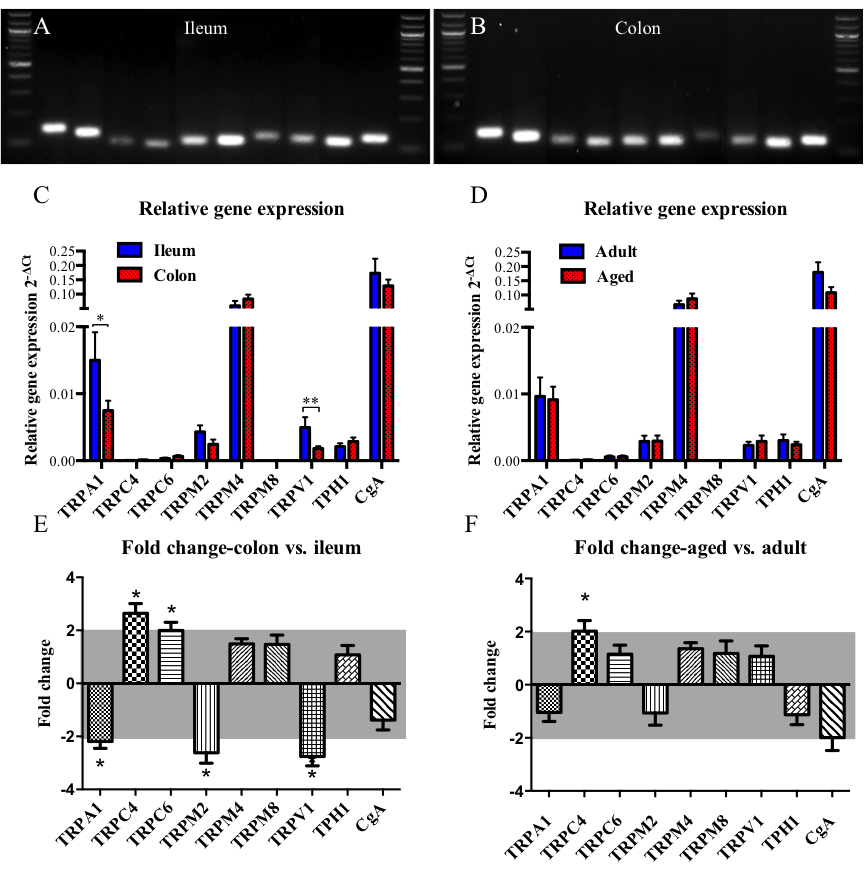


**Supplementary figure legends**

**Supplementary Figure 1. Correlation between success rate of a recording and the age and region of specimens.**

Red symbols indicate recordings that were responsive to chemical and/or mechanical stimulation, green symbols indicate the recordings that had spontaneous nerve activity but were unresponsive to chemical or mechanical stimulation and black symbols indicate the preparations that had no recordable nerve activity.

**­**

**Supplementary Figure 2. Tissue integrity and ultrastructure of recorded nerves.**

A: Transverse section of villi from a 50-year-old male ileum. The structural integrity of the mucosal epithelium is apparent demonstrating good tissue viability. B: An approximate 40 µm diameter nerve bundle running in the mesenteric attachment (the same tissue used in C). C and D show EM images of a mesenteric nerve bundle to illustrate the presence of both myelinated (C) and unmyelinated nerve fibres.

**­**

**Supplementary Figure 3. The association between mast cells and SP was not random.**

A) A superimposed image showing the spatial relationship between mast cells (in red) and SP (in green) in the colonic mucosa. B) With the same image in (A), the red channel has been flipped horizontally and superimposed on the green channel. Compared with the control in which there is close association between nerve and mast cell C), the distribution pattern in flipped image was completely broken (D). Scale bar =50 μm.

**Supplementary Figure 4. Changes in TRP channel and EC cell gene expression in the human bowel.**

A&B) Sample gels showing positive detection of all of the tested genes in the human distal ileum (56-year-old) and descending colon (62-year-old) respectively. Bands from left to right were GAPDH, TRPA1, TRPC4, TRPC6, TRPM2, TRPM4, TRPM8, TRPV1, TPH1 and CgA. C: Relative gene expression between ileum and colon. Expression of TRPA1 and TRPV1 in the colon was significant lower than in the ileum (P<0.05 vs. P<0.01; unpaired t-test, ileum vs. colon: N=5 vs. N=15). D) Relative gene expression in young (<65 years old) and aged (>65 years old) tissues. No significant difference in gene expression was seen with age.

# Supplementary methods

### **H&E staining**

## Slides were stained with *GILLS II* heamatoxylin (Surgipath, 01521E) for 5 minutes and rinsed in running water until excess dye was removed from the slide. Sections were counterstained in 1% aqueous Eosin Y in PBS for 1 minutes and rinsed in running water. After drying on a hotplate, sections were mounted with glass coverslips using DPX mounting medium.

## **Electron microscopy (EM)**

Immediately after nerve recording, the recorded nerve bundle (approximate 5mm long) was gently detached from the specimen, fixed in 3% glutaraldehyde/0.1M sodium cacodylate buffer overnight, immersed in 8% sucrose for minimal 24 hours and post-fixed in 2% aqueous osmium tetroxide for 1 hour at room temperature. Nerves were then dehydrated through graded series of ethanol (75%, 95%, 100%) for 1 hour and infiltrated in a 1:1 araldite (a mixture of CY212 and DDSA with BDMA initiator) resin/epoxy propane mixture overnight and placed into pure araldite resin for 8 hours.  Tissue blocks were prepared using fresh resin and cured for 2-3 days at 60°C oven. Tissue sections were cut at 85-90 nm onto 200 mesh thick coated copper grids using a Leica UC 6 ultramicrotome, stained in uranyl acetate and lead citrate, viewed on FEI tecnai Biotwin TEM and imaged using Gatan Orius 1000B digital camera running Gatan digital micrograph software.

## **Real-time quantitative PCR gene expression (qRT-PCR)**

The mucosa (~20mg) was removed and immediately placed in 1 mL RNAlater (QIAGEN, 76106) and stored at -20°C. Total RNA was purified using RNeasy Mini Kit (QIAGEN 74104) according to manufacturer’s instructions and reversely transcribed using High-Capacity cDNA Reverse Transcription Kit with RNase Inhibitor (Applied Biosystems, 4374966). Fluorescent probes for 7 TRP channels (TRPA1, TRPC4, TRPC6, TRPM2, TRPM4, RPM8, TRPV1) and 2 EC cell markers (CgA and TPH1) were predesigned and verified by mass spectrometry by Integrated DNA Technologies. The sequence of probes for each gene examined, ID number and expected band size are provided in supplementary Table 1. qPCR was performed using TaqMan Gene Expression Master Mix (Applied Biosystems 4369016) and BIO-RAD CFX96 Touch^TM^ Real-time system (C1000 Touch^TM^ Thermal Cycler, Bio-Rad Laboratories Ltd. Hercules, USA). Gene expression was detected as a positive band using elelctrophoresis on an agarose gel with ethidium bromide at the expected band size (Supp. Table 1). Reactions for each sample were run in duplicate. Glyceraldehyde 3-phosphate dehydrogenase (GAPDH) was used as endogenous reference gene. Data are presented as expression relative to GAPDH (1/2^ΔCt^). Samples from patients <65 years old were considered as young and samples from patients >65 years old were considered as aged.
